# Effect of Intranasal Sedation Using Ketamine and Midazolam on Behavior of 3–6 Year-Old Uncooperative Children in Dental Office: A Clinical Trial

**Published:** 2017-01

**Authors:** Majid Mehran, Sara Tavassoli-Hojjati, Nazila Ameli, Mehdi Salehi Zeinabadi

**Affiliations:** 1 Assistant Professor, Department of Pediatric Dentistry, School of Dentistry, Shahed University, Tehran, Iran; 2 Assistant Professor, Department of Pediatric Dentistry, School of Dentistry, Islamic Azad University, Tehran Branch, Tehran, Iran; 3 Assistant Professor, Orthodontic Department, School of Dentistry, Semnan University of Medical Sciences, Semnan, Iran; 4 Assistant Professor, Department of Pediatric Dentistry, School of Dentistry, Semnan University of Medical Sciences, Semnan, Iran

**Keywords:** Conscious Sedation, Ketamine, Midazolam, Administration, Intranasal

## Abstract

**Objectives:**

The aim of the present study was to compare the effects of intranasal ketamine and midazolam on behavior of 3–6 year-old children during dental treatments.

**Materials and Methods:**

In this randomized cross-over clinical trial, 17 uncooperative children requiring at least two dental treatments were selected and randomly received ketamine (0.5mg/kg) or midazolam (0.2mg/kg) prior to treatment. The other medication was used in the next visit. The children’s behavioral pattern was determined according to the Houpt’s scale regarding sleep, movement, crying and overall behavior. Physiological parameters were also measured at different time intervals. The data were subjected to Wilcoxon Signed Rank test and two-way repeated measures ANOVA.

**Results:**

The frequency of crying decreased significantly following ketamine administration compared to midazolam (P=0.002); movement of children decreased with fewer incidence of treatment interruption (P=0.001) while their sleepiness increased (P=0.003). Despite higher success of sedation with ketamine compared to midazolam, no significant differences were found between the two regarding patients’ overall behavior (P>0.05). The patients had higher heart rate and blood pressure with ketamine; however, no significant difference was found regarding respiratory rate and oxygen saturation (P>0.05).

**Conclusions:**

Ketamine (0.5mg/kg) led to fewer movements, less crying and more sleepiness compared to midazolam (0.2mg/kg). No significant differences were found between the two drugs regarding children’s overall behavior and sedation efficiency. Both drugs demonstrated positive efficacy for sedation of children during dental treatments.

## INTRODUCTION

Dental procedures, particularly local anesthesia, induce emotional stress in children, and may leave a negative impact on them [1]. Preoperative stress increases the heart rate and blood pressure due to stimulation of sympathetic, parasympathetic and endocrine systems [2]. Thus, different psychological and pharmacological methods have been introduced to decrease anxiety in children [3,4].

Premedication with safe drugs can minimize anxiety. An ideal premedication should have minimal complications, rapid onset and recovery and high acceptance by patients [5,6]. Intranasal administration of sedative drugs has been suggested as a fast, painless and noninvasive route, which has about the same onset of action as intravenous administration of drugs [7]. Midazolam and ketamine cause rapid sedation. Midazolam is an imidazobenzodiazepine, which is widely used orally and rectally in uncooperative preschool children [8–10]. Ketamine hydrochloride also has a rapid onset of action and produces well-documented anesthesia [11,12]. It has been hypothesized that preoperative sedation would be more effective in reducing children’s anxiety during local anesthesia compared to the presence of parents [13].

The literature describing the effectiveness and safety of intranasal administration of sedatives and analgesics in children has grown substantially over the past decade; however, controversy exists regarding the probable superiority of ketamine or midazolam over each other [14].

Considering the necessity of anxiety reduction before dental procedures and the great diversity in data regarding the sedative agents, the aim of the present study was to compare the sedative properties of intranasally administered midazolam with ketamine in uncooperative 3–6 year-old children.

## MATERIALS AND METHODS

Ethical approval was obtained from the Ethics Committee of the Shahed Dental School (code: 628). In this cross-over, double-blind clinical trial, 17 uncooperative [15,16] 3–6 year-old children with ASA I physical status and scale II according to the Frankl category (reluctant to accept treatment and evidence of negative attitudes) [17] were selected from the Pediatric Department of Shahed Dental School. Parents were informed about the procedure and signed informed consent forms. This clinical trial was registered in www.irct.ir (code: 16913). Selected children showed negative attitude according to the Frankel’s category and at least one dentist confirmed that they were uncooperative. They required at least two identical dental treatments including pulpotomy and restoration/stainless steel crown placement following local anesthesia. Children with upper airway infection or cognitive impairment were excluded.

After obtaining a thorough history, children were randomly assigned to receive one of the two drugs intranasally. They either received 0.2 mg/kg midazolam (Chemidaru Industrial Company, Tehran, Iran) or 0.5 mg/kg ketamine (Chemidaru Industrial Company, Tehran, Iran) in the first treatment session. In the second treatment session, scheduled with a window period of at least one week, the drugs were switched. Patients visited on odd days received midazolam while those starting treatment on even days received ketamine. Dental treatment in all patients included pulpotomy and stainless steel crown placement following local anesthesia with2% lidocaine (Pastur-Industrial Company, Tehran, Iran) with1:100.000epinephrine (Aburaihan Industrial Company, Tehran, Iran) in one of their lower quadrants.

A minimum of 6 hours [18] of NPO was suggested. All vital signs including heart rate, oxygen saturation, blood pressure and respiratory rate were recorded at baseline and monitored throughout the procedure.

A scoring system described by Houpt et al, [19] was applied for assessment of sedation. This system is comprised of the following scales:

1- Sleep scale
2- Crying scale
3- Movement scale
4- Overall scale

The level of sedation and emotional reactions including calmness and crying were estimated at baseline, during administration of anesthesia (10 minutes after administration of sedative agent) and at 5-minute intervals, 15 minutes after local anesthesia administration and at the discharge time. Moreover, parents’ experience of the procedure and side effects were questioned using a questionnaire. Physiological parameters were recorded at baseline, before sedation (T0), during administration of anesthesia (10 minutes after administration of sedative agent) (T1), 5 and 15 minutes after local anesthesia (T2 and T3) and at the discharge time (T4).

The effectiveness of sedation (Houpt’s scale) caused by the two medications at each time point was compared using Wilcoxon Signed Rank test while two-way repeated measures ANOVA was utilized to compare physiological parameters. Data were analyzed using SPSS version 22 for windows (SPSS Inc., Chicago, IL, USA) considering P<0.05 to be significant.

## RESULTS

Seventeen children (nine males and eight females) with a mean age of 4.5±0.9 years were studied. The mean weight of children was 16.2±3.6 kg (range 10.5–24 kg).

Heart rate and blood pressure significantly increased following ketamine administration at all time points.

Sleep scale: More fully awake children were found following midazolam sedation (64.7%) as compared to ketamine (0) at the time of local anesthesia administration (P=0.003). While, during restorative treatment and at the discharge time, this difference was not significant (Table 1).

**Table 1:** Sleep scores following ketamine/midazolam administration at different time points

**Time point**	**Local anesthesia administration**			**5 minutes after anesthesia administration**			**15 minutes after anesthesia administration**		

**Drug**									

	**1**	**2**	**3**	**1**	**2**	**3**	**1**	**2**	**3**
	
**Ketamine**	0	88.2%	11.8%	5.9%	82.4%	11.8%	64.7%	29.4%	5.9%
**Midazolam**	64.7%	29.4%	5.9%	11.8%	88.2%	0	35.3%	58.8%	5.9%
	
**P-value**	0.003			0.180			0.157		

Crying: In most children in both visits, the crying score was recorded as intermittent or no crying (Table 2). Chi square test showed that except for the time of anesthesia administration (P=0.002), the differences in crying score were not significant between the two medications.

**Table 2:** Crying scores following ketamine/midazolam administration at different time points

**Time point**	**Local anesthesia administration**				**5 minutes after anesthesia administration**		**15 minutes after anesthesia administration**											

**Drug**																		

	**1**	**2**	**3**	**4**	**2**	**3**	**4**	**1**	**2**	**3**	**4**
	
**Ketamine**	0	5.9%	29.4%	64.7%	11.8%	41.2%	47.1%	0	23.5%	58.8%	17.6%
**Midazolam**	5.9%	29.4%	58.8%	5.9%	5.9%	76.5%	17.6%	5.9%	17.6%	41.2%	35.3%
	
**P-value**	0.002				0.166		0.660											

Movement: In most children, movement did not lead to interruption of dental treatment although a significant difference was observed at the time of local anesthesia administration (Table 3).

**Table 3:** Movement scores following ketamine/midazolam administration at different time points

**Time point**	**Local anesthesia administration**			**5 minutes after anesthesia administration**			**15 minutes after anesthesia administration**		

**Drug**									

	**2**	**3**	**4**	**2**	**3**	**4**	**2**	**3**	**4**
	
**Ketamine**	5.9%	76.5%	17.6%	5.9%	29.4%	52.9%	5.9%	47.1%	47.1%
**Midazolam**	70.6%	29.4%	0	0	52.9%	35.3%	11.8%	17.6%	70.6%
	
**P-value**	0.001			1.00			0.414		

During local anesthesia administration, the children sedated with intranasal midazolam demonstrated significantly more movement (P=0.001) than those sedated with ketamine.

Overall behavior: Most children exhibited good or very good behavior in both visits with just one poor behavior 15 minutes after restorative treatment (Table 4). Although ketamine sedation resulted in more favorable behavior, no significant differences were observed between two dental visits (P>0.05). Perioperative side effects including oxygen desaturation (SpO2 < 90%), disruptive movement, nausea, vomiting and nasal discomfort were also noted. The most prevalent side effects of nasal administration of midazolam and ketamine were found to be nasal discomfort (38.2%) and vomiting (35.3%), respectively.

**Table 4:** Overall scores following ketamine/midazolam administration at different time points

**Time point**	**Local anesthesia administration**				**5 minutes after anesthesia administration**				**15 minutes after anesthesia administration**			

**Drug**												

	**2**	**3**	**4**	**5**	**2**	**3**	**4**	**5**	**2**	**3**	**4**	**5**
	
**Ketamine**	5.9%	17.6%	70.6%	5.9%	5.9%	23.5%	41.2%	29.4%	5.9%	23.5%	41.2%	29.4%
**Midazolam**	5.9%	58.8%	35.3%	0	0	35.3%	58.8%	5.9%	0	35.3%	58.8%	5.9%
	
**P-value**	0.052				0.464				0.564			

## DISCUSSION

The aim of the present study was to compare the sedative properties of intranasal administration of midazolam and ketamine in uncooperative 3–6 year-old children. It is well-known that preoperative anxiety in children would result in subsequent behavioral problems and consequences such as bad dreams [20].

Intranasal drug administration is a relatively new route of drug delivery and has been reported to produce safe, effective and rapid sedation [21]. The present study showed that both ketamine and midazolam intranasal administration produced acceptable sedation with equal effects. We did not compare the drugs with placebo as it has been reported that they are superior to placebo [22–24]. The dosage of ketamine used in our study was 0.5 mg/kg, since Hosseini Jahromi et al, [25] suggested that increasing the dose of intranasal ketamine would result in less sedation and a low dose of 0.5mg/kg might be appropriate with less side effects. Moreover, the dosage of applied midazolam was 0.2 mg/kg as Ozen et al, [26] reported that the highest success rate of sedation is observed following intranasal use of 0.2mg/kg midazolam followed by 0.75mg/kg orally.

Kazemi et al, [1] reported that 0.2mg/kg intranasal midazolam and 0.5mg/kg ketamine in 2–5 year-old children lead to easier separation of children from their parents, which is comparable to our findings. Similarly, Lightdale et al, [27] compared the sedative effects of ketamine and midazolam/fentanyl in children undergoing gastrointestinal endoscopy and reported that children sedated with ketamine showed about the same movement score as patients sedated with midazolam/fentanyl.

Conversely, Singh et al, [28] performed a study to compare the sedative effects of oral midazolam with other sedative agents in children and demonstrated that oral midazolam produced the best level of sedation. Bahetwar et al, [29] compared the sedative effects of ketamine, midazolam and their combination and concluded that the difference between the overall success rates of intranasal ketamine and midazolam is not statistically significant and both are effective and safe to induce moderate sedation for dental procedures in children. Differences in the design and protocols of drug administration could result in variable results. The most prevalent side effect of intranasal administration of midazolam was found to be nasal discomfort (38.2%), which is similar to the findings of Ljungman et al, [30] who reported a prevalence of 45%, which could even lead to sample dropout. On the other hand, the most common side effect following ketamine administration was vomiting (35.3%), which is consistent with the findings of Holloway et al, [31] who reported a 14% frequency of vomiting after intramuscular administration of ketamine. However, this postoperative side effect was transient. In our study, ketamine resulted in a significant increase in blood pressure and heart rate, which could be explained by the fact that it is a cardiopulmonary stimulant [32]. However, these changes did not lead to interruption of treatment.

Conversely, Tanaka et al, [33] reported no significant difference in heart rate and blood pressure following rectal administration of ketamine and midazolam. This could be explained by more rapid and greater absorption of drugs through nasal route.

## CONCLUSION

The results of the present study demonstrated that adequate sedation is induced by both midazolam and ketamine. Ketamine administration produced marginally higher levels of blood pressure and heart rate.
